# Effect of Heat Treatment States of Feedstock on the Microstructure and Mechanical Properties of AA2219 Layers Deposited by Additive Friction Stir Deposition

**DOI:** 10.3390/ma16247591

**Published:** 2023-12-11

**Authors:** Ming Zhang, Xianjue Ye, Yidi Li, Hui Wang, Ruilin Lai, Yunping Li

**Affiliations:** State Key Lab for Powder Metallurgy, Central South University, Changsha 410083, Chinayexianjue@csu.edu.cn (X.Y.); liiidi@csu.edu.cn (Y.L.); wanghuiii@csu.edu.cn (H.W.); lairuilin@csu.edu.cn (R.L.)

**Keywords:** additive manufacturing, 2219 Al-Cu alloys, friction stir additive deposition, microstructure, mechanical properties

## Abstract

This study is the first to research the microstructure and mechanical properties of the workpiece after additive friction stir deposition (AFSD) of the feedstock at different heat treatment stages. AA2219 aluminum alloys with three different heat treatment stages were selected as the feedstock, and alloys with dense structure were successfully prepared by the additive friction stir deposition AFSD process. Experimental results show that AFSD exhibits an excellent ability to refine grains and improve the uniform distribution of precipitates in the second phase, thereby improving the plasticity of AA2219 alloy after the AFSD process. Because of the continuous dynamic recrystallization (CDRX) in the AA2219 alloy during AFSD, the grain size after the AFSD process is independent of the initial feedstock grain size for three samples. The equilibrium phase (θ) size is genetically related to the initial size of the second-phase particles in the feedstock. Due to grain refinement and dislocation strengthening, the yield strength of AA2219-casting increased significantly from 79.8 MPa to 124.1 MPa after AFSD. The yield strength of the AA2219-T4 decreases slightly from 151.8 MPa to 140.4 MPa after AFSD. The precipitation of the second phase leads to a decrease in solid solution strengthening and dislocation strengthening. However, grain refinement strengthening partially offsets this reduction. The yield strength of AA2219-T87 decreased from 398.5 MPa to 147.2 MPa after AFSD. As such, grain refinement strengthening and solid solution strengthening by the AFSD process are much smaller than the yield strength lost by precipitation strengthening and dislocation strengthening.

## 1. Introduction

The 2xxx series Al-Cu alloys are commonly used in aerospace structures due to their relatively high fracture toughness and strength to weight ratios [1,2,3,4]. Among the 2xxx series alloys, the aluminum alloy AA2219 is known for its excellent low temperature properties, enhanced strength to weight ratio and tolerance to stress corrosion cracking [5,6,7]. Therefore, it is widely used to make rocket fuel storage tanks [8,9,10]. However, traditional melt-based additive manufacturing (AM) methods involve the melting and solidification of materials, which can severely affect the mechanical properties of these alloys through the formation of coarse particles, cracks, and pores. In recent decades, AM has attracted much attention due to its inherent advantages such as a short processing time and high feedstock utilization [11]. Depending on the heat source, additive manufacturing processes can be divided into electron beam additive manufacturing, laser additive manufacturing, wire additive manufacturing and additive friction stir deposition (AFSD). AM technology is reliably used to produce various Al-Cu alloys—Al-5.23Cu-0.8Li [12], Al-4.43Cu-1.31Zr [13], Al-3.5Cu-1.5Mg [14], etc. AFSD is a new type of solid-state additive technology suitable for large-scale additive manufacturing, coating [15], restoration of metals [16,17] and composites [18]. The Aeroprobe company firstly researched the technology of AFSD and named its technology and equipment MELD to distinguish it from the general AFSD.

AFSD is a process that involves softening and depositing material onto substrates using frictional heat and plastic deformation. In order for the materials to be deposited successfully, the temperature range should reach 0.6 to 0.9 fold the melting temperature (Tm) [19]. During the AFSD process, there are changes in temperature which inevitably result in changes in the substructure of the material, such as grain size, dislocation density, and secondary phase. Understanding the influence of these microstructure changes on the mechanical properties of AA2219 is crucial. It is important to thoroughly comprehend the microstructural variations in the AFSD process to gain a better understanding of its fundamentals. It is worth noting that AFSD was developed based on friction stir welding (FSW), and both processes have similar thermal histories, as reported by David et al. [20]. Therefore, it is necessary to discuss previous studies on FSW to shed light on the deposition mechanism during the additive process of AA2219. Previous studies have examined the microstructural effects of FSW on different tempers of AA2219 [21,22,23]. In the FSW process, the material in the weld nugget experiences high temperatures and shear forces, causing the formation of fine equiaxed grains. When the feedstock is in an aged state, the strength and toughness may be reduced due to the solid solution of the metastable precipitated phase in the matrix. Due to the swift cooling rate, the matrix retains this metastable phase; however, larger metastable phases undergo a transition to a state of stability. Wang et al. [24] observed only a few equilibria phase θ precipitates in the nugget zones (NZ). The Lowest Hardness Zone (LHZ) has a lower density of equilibrium phase compared to the feedstock. During the SR-FSW process, a thermocouple is placed in the central area of the board to measure the temperature. Zhou et al. [25] found that the welding speed increased from 150 mm/min to 450 mm/min and the temperature decreased from 454 °C to 420 °C. Wedge et al. [26] determined that the temperature of the stirring zone in the FSW process could reach approximately 550 °C, which is close to the eutectic temperature of AA2219. For temperatures below 400 °C, the dissolution rate of Al_2_Cu in the Al matrix remains relatively stable, but it increases sharply when the temperature is between 400 °C and 550 °C. Once the temperature of the stirring zone approaches the eutectic temperature, the second-phase Al2Cu will no longer exist, and the alloy becomes a solid solution as the copper diffuses back into the matrix. Rivera et al. [27] were the first to use the MELD process to deposit six layers of AA2219 alloy on an AA2219-T6 plate. The selected feedstock was 2219-T851, and each layer had a thickness of 1 mm. The tensile strength of each layer after the addition was found to be lower than that of AA2219-T87. TEM analysis revealed similarities to FSW [28], but no θ′ phase was observed in the deposited layer. In their study, Wedge et al. [29] focused on determining the best process parameters for the production of AA2219 through the MELD process. They used 2219-T87 as the selected feedstock and found that the grains of AFSD material were 5.5-fold smaller compared to the feedstock. They observed that the θ′ and θ″ phases were basically absent, while the feedstock still contained a significant amount of primary strengthening phase. The researchers exclusively concentrated on the redissolution occurrence of the fortified phase (θ′) and disregarded the alterations in the equilibrium phase. However, the periphery of the equilibrium phase is often the origin of cracks and plays a pivotal role in the deterioration of material properties. Furthermore, the existing research methods do not allow in situ observation of dissolution and precipitation behavior of the precipitation phase, making it challenging to accurately control mechanical behavior of the workpiece after the AFSD process.

FSW is capable of welding feedstock in different heat treatment states. However, AFSD typically selects feedstock in the same state to ensure isotropic and uniform workpieces. It is important to note that different heat treatment states result in different initial microstructures. The question arises whether these different initial microstructures have an impact on the mechanical properties after AFSD. To obtain artifacts that meet the usage requirements, it is crucial to carefully select the appropriate material as the feedstock. Surprisingly, previous research reviews have neglected to investigate and analyze the effect of heat treatment conditions of aluminum alloys on the microstructure changes that occur during AFSD. Research on this topic is important for understanding and revealing the AFSD mechanical properties changes in aluminum alloys. This study selected three aluminum alloys (AA2219-casting, AA2219-solution treatment and AA2219-T87) with different heat treatment states as experimental materials for AFSD. The characterization and testing of the microstructure and mechanical properties were conducted before and after AFSD, respectively. Combining test results, researchers try to connect the microstructure and mechanical properties before and after AFSD to provide insights for the selection of feedstock in subsequent production.

## 2. Materials and Methods

### 2.1. Material Preparation and Experimental Details

The nominal chemical composition (wt.%) of the AA2219 alloy is shown in Table 1, the compositions of the above alloys are all within the scope of this table.

AA2219-casting: Li et al. [30] previously reported the manufacturing process of a large AA2219 alloy casting ingot, which was used as the test material in this study. The ingot had a chemical composition of Al-6.2Cu-0.36Mn-0.11Zr-0.1V-0.10Fe-0.06Si-0.01Mg-0.10Zn-0.05Ti (wt%). Central South University, China, supplied the casting ingot, which had dimensions of Ø650 mm × 4800 mm. To prepare the samples, the casting ingot was pre-cut into square bars of 11 mm × 11 mm × 200 mm using machining. The surface of the bars was then peeled and processed to obtain a smooth surface, resulting in square rods measuring 10 mm × 10 mm × 200 mm.

AA2219-T87: The test material used in this study was a large AA2219 alloy plate measuring 50 mm × 1200 mm × 500 mm. After solution treatment, the cold working process with a reduction rate of approximately 7% was applied to a section of the plate, and then the artificial aging treatment was carried out. The chemical composition of the alloy was Al-6.2Cu-0.29Mn-0.13Zr-0.08V-0.13Fe-0.04Si-0.01Mg-0.01Zn-0.04Ti (wt%). The alloy plate was purchased from Nannan Aluminum, located in Nanning, Guangxi, China. The as-received material was then machined into square rods measuring 10 mm × 10 mm × 200 mm along the rolling direction.

AA2219-T4 (solution treatment): The plate of AA2219-T87 underwent solid solution treatment at 535 °C for 70 min and was then quenched in water at room temperature. The surface was then peeled and processed into a square rod measuring 10 mm × 10 mm × 200 mm.

**Figure 1:**
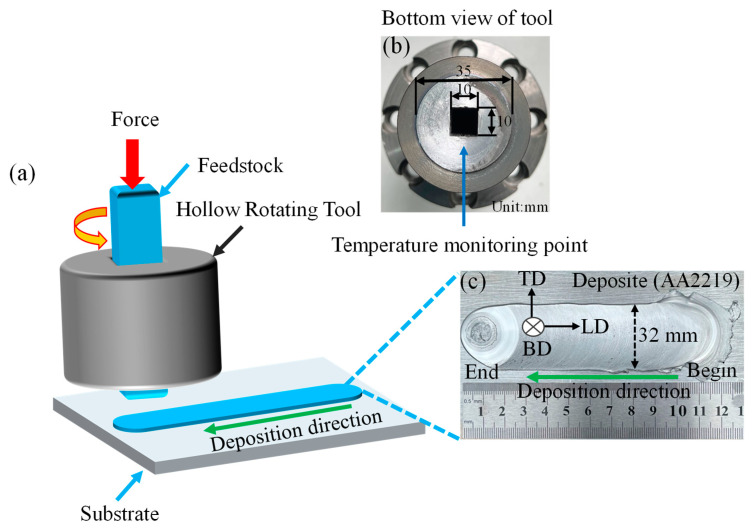
(**a**) Schematic of the AFSD process; (**b**) the cutter head used in the AFSD process and the temperature measurement position of the built-in thermocouple; (**c**) photograph of AA2219 alloy deposited with a single layer.

Using a FRC002 machine (Central South University, China.), the solid-state material deposition process was conducted as shown in Figure 1a. A 10 mm × 10 mm square rod feedstock of AA2219 alloy was deposited on a 6 mm-thick AA2219-T87 substrate. Before deposition, graphite pre-coating was applied to the feedstock to minimize friction between the tool and the feedstock. Furthermore, the oxide layer was eliminated by grinding the substrate using 600 # sandpaper. The feedstock was fed through a rotating tool with a diameter of 36 mm, which had a flat surface as depicted in Figure 1b. To monitor the temperature change in the material during AFSD, we drilled on the side of the cutter head to accommodate the thermocouple, as depicted in Figure 1c. The width of the deposited single layer of AA2219 was measured to be 32 mm, with an estimated thickness of approximately 2 mm. At the beginning of deposition, a pre-heating process of approximately 50 s was required, resulting in noticeable material stacking and flash edges. Once the heat generation process was completed, the tool head started moving along the additive direction, leaving a keyhole at the end. Henceforth, we will designate the build direction as BD, the transverse direction as TD, and the longitudinal direction as LD.

**Figure 2:**
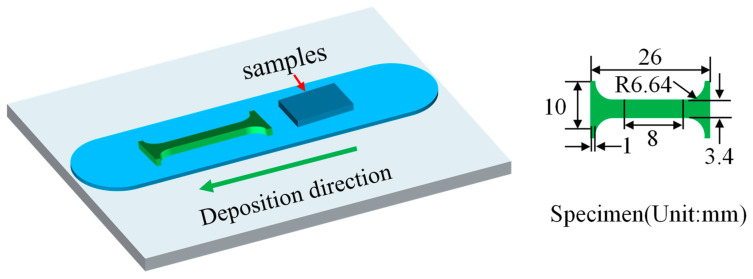
Sampling schematic, sampling area and dimensions of the stretched sample.

The proportion of the feed rate (F) to plane velocity (V) is a crucial factor in achieving high-quality print results. Prior to manufacturing the AA2219 build, a process parameter optimization study was conducted. Based on the volume calculation, it was determined that when F is set to 100 mm/min, the ideal range for V/F is 1.25–1.75. This range ensures that the deposited material exhibits excellent surface quality and minimizes the occurrence of defects like flash. Therefore, in this research, the V/F ratio was set to 1.5 (F = 100 mm/min, V = 150 mm/min, ω = 400 RPM). During the deposition phase, the tool head remained stationary while generating heat. For this phase, F was set to 20 mm/min and ω was set to 400 RPM. It is important to highlight that the distance separating the tool head and substrate measured approximately 0.85 mm, which is lower than the ultimate deposition thickness of 2 mm. This phenomenon was identified by Perry et al. as the outcome of the uncontrolled upward movement when the substance leaves the depositional area on the posterior side [31]. The specimens were cut parallel to the LD and length direction of the square bar to allow examination of the upper surface. Additionally, samples shaped like dog bones were cut in parallel to the LD and length direction of the square bar to perform tensile testing. The dimensions of the tensile sample are illustrated in Figure 2, the size of the tensile test specimen conforms to GB-T 228.1-2010 [32]. Using a WDW-5 (Jinan Chuanbai Instrument Equipment Co., Ltd., Jinan, China) universal test machine, the mechanical properties were assessed at a crosshead speed of 0.5 mm/min.

### 2.2. Material Characterization

An optical microscope (OM), a scanning electron microscope (SEM), an electron backscattered diffraction (EBSD), a transmission electron microscope (TEM), and X-ray diffraction (XRD) were utilized to observe the microstructure of the specimens. The microstructure observation specimens underwent polishing using an MP-2B polishing machine. For optical microscope observation, in addition to mechanical polishing, chemical etching for approximately 5–20 s was performed with Keller’s reagent. The SEM observation, BES observation, and EBSD analysis were conducted on a Quanta 650 FEG (Rock Hill, SC, USA). For EBSD observation, the specimens were prepared by electro-polishing using a solution of 90% alcohol and 10% perchloric acid at 20 V. The EBSD scan step size was 0.3–0.5 μm and the operating voltage was 20 kV. TEM specimens were prepared using a twinjet electro-polishing technique, and the solution employed for electro-polishing consisted of 25% nitric acid and 75% methanol. The process was conducted at a voltage of 14 V and a temperature of −30 °C. The scanning TEM (STEM) map was obtained by Talos F200X G2 (FEI, Brno-Královo Pole, Czech Republic) at 200 kV, the size and spatial dispersion of precipitated phase were imaged by a high-angle annular dark field (HAADF) detector. XRD scanning was carried out using the D/Max 2550VB (Rigaku, Tokyo, Japan) equipment, and a Cu-Kα probe with a wavelength of 0.154 nm was used. The XRD test had a step size of 0.02° and a scanning speed of 3°/min. The scanning range of 2θ was from 20 to 85°. We calculated the average size (AS) and average fraction (AF) of Al_2_Cu (θ phase) using Image J (Java 1.8.0), and θ phase according to size can be classified into coarse second-phase particles (CSPPs) and fine second-phase particles (FSPPs).

## 3. Results

### 3.1. Microstructure before and after the AFSD Process

**Figure 3:**
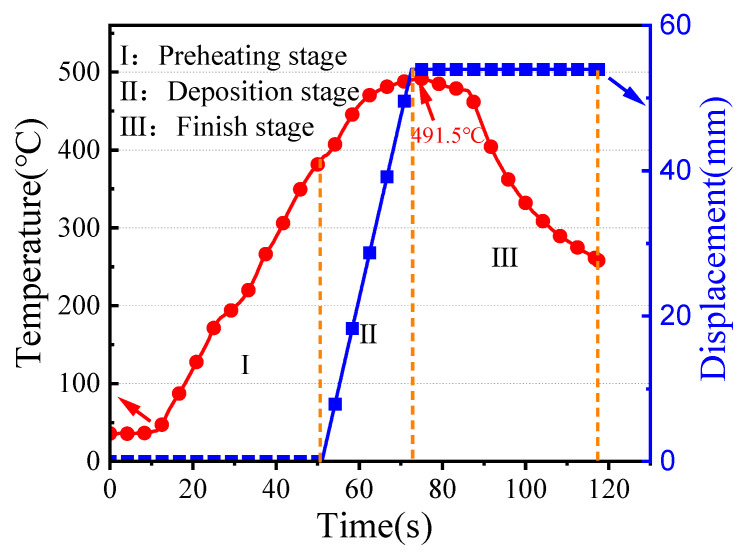
Temperature and displacement evolution during the AFSD process.

As shown in Figure 3, the temperature profile at the end face of the tool head with deposition time was recorded. The deposition process consists of three stages: the pre-heating stage, the deposition stage, and the finishing stage. The highest temperature recorded during the stable deposition stage is 491.5 °C, which is also the temperature experienced by the deposition layer.

**Figure 4:**
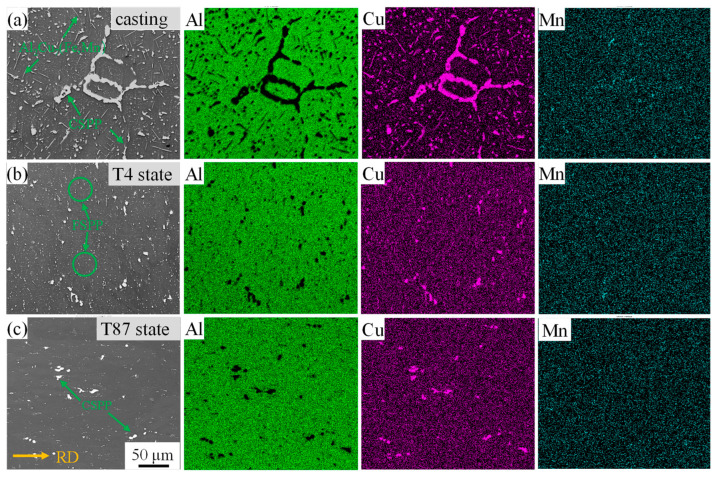
The SEM micrograph of the feedstock and corresponding elemental mappings. (**a**) Casting, (**b**) T4 state, and (**c**) T87 state.

Figure 4 presents the SEM micrograph of the feedstock and the corresponding elemental mappings. In the casting process of AA2219, composition segregation occurs, and large agglomerated particles (CSPPs) and needle-like precipitates Al_7_Cu_2_(Fe, Mn) can be observed in Figure 4a [33]. CSPPs were also observed in AA2219-T4 and AA2219-T87 (Figure 4b,c). However, after a long-time homogenization treatment, composition segregation disappeared and the CSPP size decreased significantly. The surface scan results of elemental mappings support this consequence.

**Figure 5:**
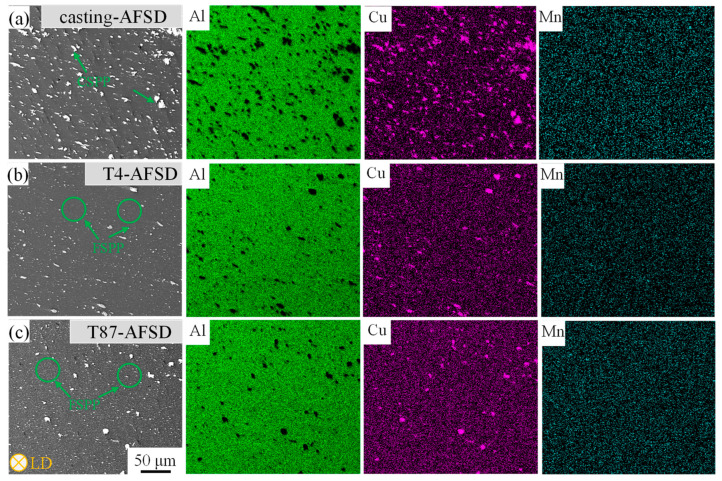
The SEM micrograph of the deposit and corresponding elemental mappings. (**a**) Casting-AFSD, (**b**) T4-AFSD, and (**c**) T87-AFSD.

Figure 5 presents the SEM micrograph of the deposit and corresponding elemental mappings. The size of Al_2_Cu particles in the AA2219-casting was observed to be broken and reduced without agglomeration after the AFSD process, as depicted in Figure 5a. This observation is further supported by the energy elemental mappings. Additionally, when compared to AA2219-T4, the T4-AFSD exhibited a substantial increase in the production of FSPPs, as shown in Figure 5b. Similarly, Figure 5c illustrates a notable increase in the number of FSPPs for T87-AFSD.

**Figure 6:**
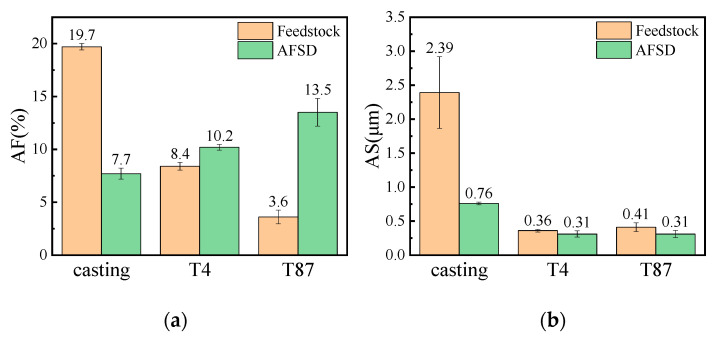
Histogram of AF and AS of θ phase in AA2219 under different processing states: (**a**) area fraction and (**b**) average size.

To demonstrate the changes in size and content of the equilibrium θ phase in AA2219 alloy before and after the AFSD process, the AF and AS were calculated and the results are shown in Figure 6a and Figure 6b, respectively. It was observed that the AF and AS underwent significant changes in AA2219-casting after the AFSD process. The AF of the precipitates decreased from 19.7% to 7.7%, and the AS decreased from 2.39 μm to 0.76 μm. In AA2219-T4 after the AFSD process, the AF increased from 8.4% to 10.2%, and the AS decreased from 0.36 μm to 0.31 μm. In T87-AFSD, the AF increased from 3.6% to 13.5%, and the AS decreased from 0.41 μm to 0.31 μm.

**Figure 7:**
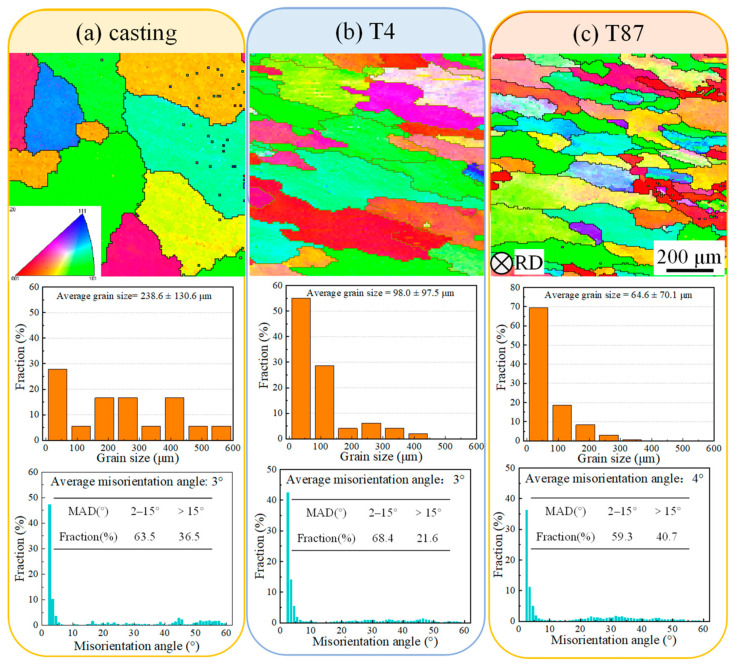
EBSD results of feedstock under different conditions. (**a**) Casting, (**b**) T4 state, and (**c**) T87 state.

**Figure 8:**
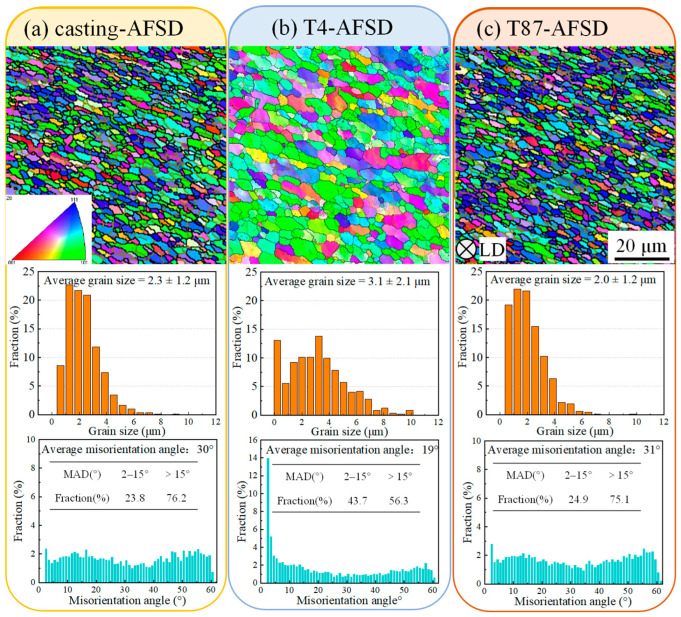
EBSD results of feedstock after AFSD. (**a**) Casting-AFSD, (**b**) T4-AFSD, and (**c**) T87-AFSD.

To analyze the microstructure of the AA2219 alloy after AFSD, this study utilized EBSD to examine the distribution of grain sizes and grain boundary misorientations. These analyses are depicted in Figure 7 and Figure 8.

The results revealed that the original grain size or shape was transformed into equiaxed grains after the AFSD process, with an average grain size ranging from 2.3 μm to 3.1 μm. No significant grain texture was observed, indicating recrystallization in the feeding materials due to the elevated temperature (measured at approximately 492 °C) and plastic deformation [27]. The statistical analysis of grain orientation showed that 76.2% and 75.1% of the high-angle boundaries (HABs) were present in casting-AFSD and AFSD-T87, respectively, indicating a high degree of recrystallization. Furthermore, T4-AFSD exhibited a relatively low percentage of low-angle boundaries (LABs) after the AFSD process.

**Figure 9:**
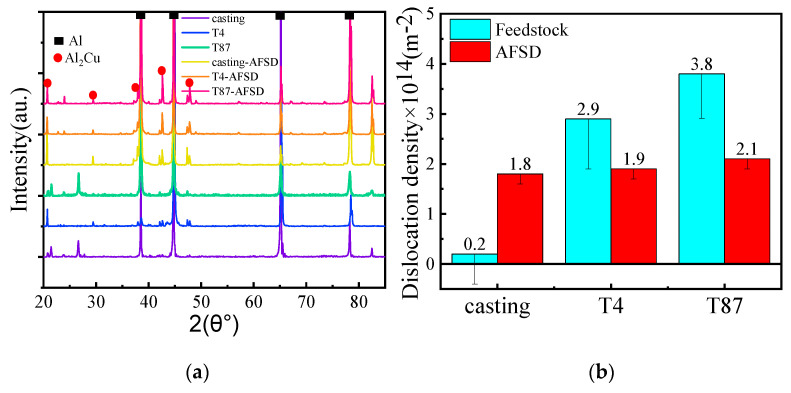
AA2219 alloy under different processing conditions. (**a**) XRD patterns and (**b**) dislocation density calculation results.

The XRD patterns for different specimens (before and after the AFSD process) were shown in Figure 9a. The dislocation density can be evaluated using the following equation, based on the XRD patterns [34]:

(1) $$\frac{\beta \cos\theta }{\lambda }=\frac{0.9}{D}+\frac{2\varepsilon \sin\theta }{\lambda }$$

(2) $$\rho =\frac{2\sqrt{3}\varepsilon }{Db}\;$$

The half-maximized height of the peak width in the X-ray diffraction pattern is denoted by *β*, and the Cu wavenumber (0.1542 nm) of the *K_α_* radiation is denoted by *λ*. The grain size is represented by *D*, the lattice strain by *ε*, and the Bragg angle by θ. It should be noted that the grain size of the samples is well over 100 nm, so its influence on the peak broadening is not readily apparent. Consequently, the dislocation density can be evaluated using Equations (1) and (2), and the corresponding results are presented in Figure 9b. The dislocation density of the metal work hardened state is generally 10^13^~10^14^ m^−2^, the dislocation density in AA2219-casting is approximately 2.0 × 10^13^ m^−2^, and it significantly increased to 1.8 × 10^14^ m^−2^ after the AFSD process; in the other two samples after the AFSD process, dislocation density decreased from 2.9 × 10^14^ m^−2^ and 3.8× 10^14^ m^−2^ to 1.9× 10^14^ m^−2^ and 2.1 × 10^14^ m^−2^, respectively.

**Figure 10:**
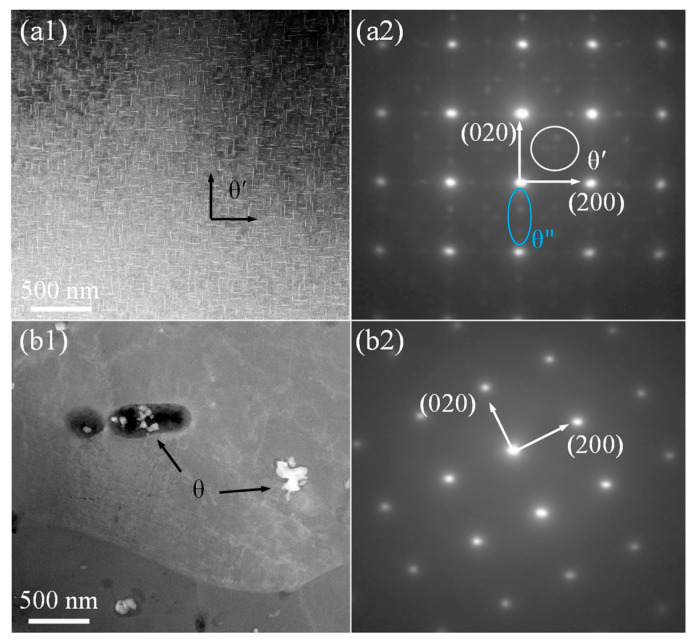
STEM images and corresponding SAED pattern of AA2219 alloy: (**a1**,**a2**) T87 and (**b1**,**b2**) T87-AFSD.

In Figure 10, STEM images and the corresponding SAED pattern of AA2219-T87 before and after the AFSD process are presented. The images in Figure 10(a1) show the presence of precipitates, specifically the θ′ phase, with an AF of 20%. These precipitates have a length ranging from 45 to 100 nm and an average width (AW) of 5 nm. The confirmation of the precipitates as θ′ phase is supported by the visible bright spot that appears at the {001}_Al_ position in the corresponding SAED pattern shown in Figure 10(a2). In addition, the discontinuous stripes in the SAED pattern along {020}_Al_ indicated that precipitates include the θ″ phase. Compared with Figure 10(a1), some disc-shaped precipitates—AF 3.0%, length 50–200 nm, width 15 nm—were observed in Figure 10(b1), with no observed θ′ phase, and the corresponding SAED pattern in Figure 10(a2) has no significant bright spots observed at the {001}_Al_ position.

**Figure 11:**
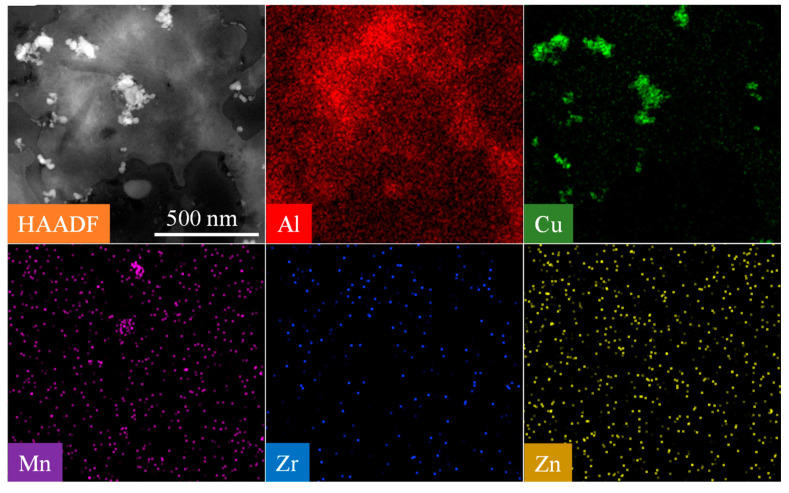
HAADF-STEM images and corresponding EDS mappings of T87-AFSD.

Figure 11 shows HAADF-STEM images and corresponding EDS mappings of T87-AFSD. Some disc-shaped precipitates were observed in HAADF-STEM images, Through EDS mapping, it was found that the Cu element was segregated, and other elements (Mn, Zr, Zn) were evenly distributed in the aluminum matrix, which further confirmed that the disc-shaped precipitation was equilibrium θ phase.

### 3.2. Mechanical Properties and Fracture

**Figure 12:**
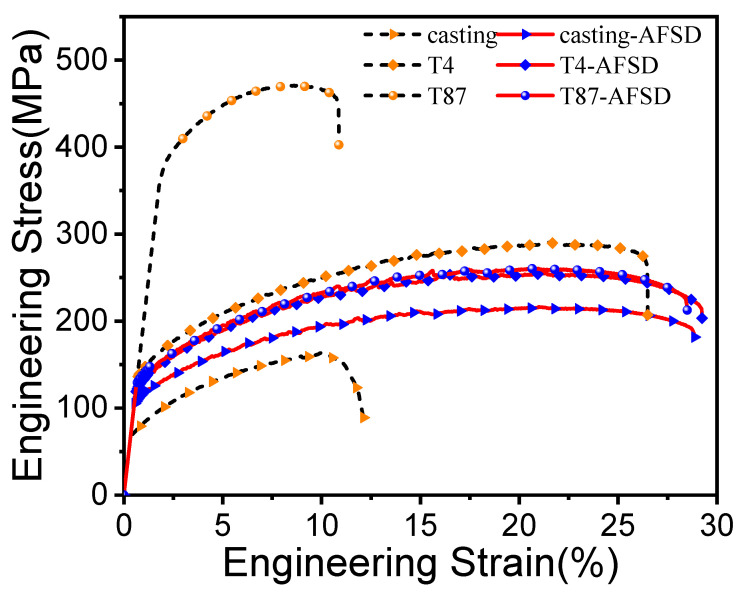
The engineering stress–strain curves for AA2219 alloy under different processing conditions.

Figure 12 presents the engineering stress–strain curves for AA2219 under different processing conditions. To ensure the consistency of the experimental results, each group of tensile tests was performed at least three times, and tolerance calculations were conducted. The statistical results are shown in Table 2.

**Figure 13:**
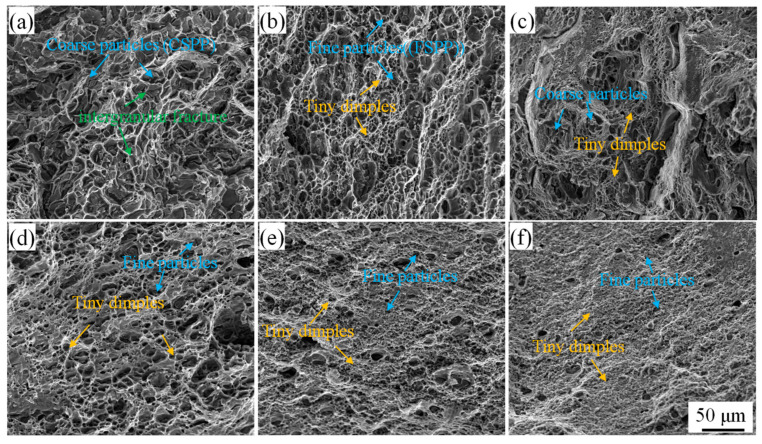
Fracture organization of AA2219 in different processing states: (**a**) casting; (**b**) T4; (**c**) T87; (**d**) casting-AFSD; (**e**) T4-AFSD; (**f**) T87-AFSD.

To gain a deeper understanding of the improvement mechanism in the mechanical properties after the AFSD process, it is important to compare and analyze the fracture behaviors of different feedstock before and after the AFSD undergoing process. Figure 13 presents SEM micrographs of the fracture surfaces of AA2219 alloy in various processing states. On the fracture surface of the casting (see Figure 13a), CSPPs can be observed, and the material fracture occurs through intergranular fracture. The agglomeration zone of CSPPs serves as the initiation site and easy propagation channel for cracks, leading to poor plasticity (10.9%) in casting. However, after the AFSD process, no significant CSPPs were found on the fracture surface of the casting-AFSD specimens. The second-phase particles are more diffuse, and the presence of dimples is more pronounced, as shown in Figure 13d. In Figure 13b, it can be observed that there is no presence of CSPPs, and compared to Figure 13a, most of the second-phase particles have dissolved. Consequently, tiny dimples are formed, resulting in an enhancement of plasticity. Furthermore, after the AFSD process, T4-AFSD exhibits more FSPPs, more pore nucleation sites, and a greater number of continuous tiny dimples, as depicted in Figure 13d. Figure 13c shows that 2219-T87 contains both CSPPs and numerous dimples, attributed to the peak aging treatment and the abundance of second-phase particles, leading to higher yield strength but low ductility. However, after the AFSD process, the dimples become smaller and more continuous, leading to an increase in material plasticity.

## 4. Discussion

According to the results, the material experienced significant deformation at high temperatures during AFSD. This process also led to noticeable microstructural changes, such as grain refinement and a relative change in dislocation density. The existence of microstructural changes and second-phase particles caused the AA2219 alloys, which underwent three different heat treatments, to exhibit varying mechanical properties after deposition.

### 4.1. Microstructural Changes

During AFSD, the feeding materials experienced plastic deformation at high temperatures, which caused recrystallization and a decrease in dislocation density. Continuous dynamic recrystallization is a process where dislocations gradually accumulate, annihilate, and recombine during deformation and dynamic recovery. This process leads to the formation of sub-grains. The size of the dynamically recrystallized grains (*D_d_*) is determined by the Zener–Hollomon parameter (*Z_H_*), which is dependent on the temperature (*T*) and the strain rate ($\dot{\varepsilon }$). The relationship between *D_d_*, *Z_H_*, *T*, and $\dot{\varepsilon }$ can be described by the following formulas [35,36]:

(3) $$D_{d}=c{Z_{H}}^{-n_{d}}\;$$

(4) $$Z_{H}=\dot{\varepsilon }exp(\frac{Q_{def}}{RT})$$

In which *R* is the gas constant, *c* and *n_d_* are constants, $\dot{\varepsilon }$ is the strain rate and *Q_def_* is the activation energy for hot distortion. According to Equations (3) and (4), the grain size after AFSD depends on the heat input by the tool head [37] and the strain rate. Since the processing parameters (tool head speed, traverse speed, and height of the tool from the substrate) for three groups were set to be critical, the heat input is assumed to be the same for all groups. There is a certain difference in grain size (see Figure 8), which may be caused by a little difference in heat output due to differences in initial material accumulation, substrate surface state, feedstock surface state, etc. In the process of this research, the grains in the casting-AFSD and T87-AFSD are relatively close, and it is considered that the initial condition control is relatively similar. According to Hillert [38], the final grain size in a material is influenced by the ability of a second-phase particle to cut off when the grain boundary shifts. The grain size of T4-AFSD is larger than that of the other two sets, which proves the loss of the hindering effect on grain boundary migration, resulting in grain growth.

### 4.2. Second-Phase Particles Dissolution and Precipitation Behavior

AA2219 alloy is a precipitation-strengthened alloy, where a series of metastable phases precipitate from a supersaturated solid solution during cooling. The Al-Cu precipitation process follows a sequence of steps at relatively low aging temperatures: Al_ss_→GP I zones→θ″(GP II zones) →θ′→θ, θ″(GP II zones) and θ′ are the primary strengthening precipitates phases, it is coherent or semi-coherent with the Al matrix, which obstructs the movement of dislocations, resulting in enhancement of the strength. However, the equilibrium θ is incompatible with the Al matrix [39]—the smaller the size, the better the plasticity of the material.

During the AFSD process, the feedstock flows extensively at high temperatures, second phase changes with material flow and temperature. Papazian et al. [40] proposed the dissolution temperature of θ″ phase is 100–200 °C, the θ′ phase formation temperature is 200–300 °C, θ′ the phase dissolution temperature is 300–400 °C, and the θ phase formation temperature is 400–450 °C. As can be seen from Figure 10(b1), no metastable precipitates (θ′ and θ″) are present but only the θ phase was observed. This is consistent with recent studies [27,41], in which the temperature during the AFSD process exceeds 500 °C [20], and temperature measurement results of the cutter head also verified this. This can be rationalized for two reasons. First, at temperatures above 480 °C, the metastable phases (θ′ and θ′) average to the equilibrium phase. Second, if the temperature of the solvent is above 513 °C, the metastable phase can dissolve into the matrix and subsequently precipitate as the θ phase during slow cooling. Determining which of the above mechanisms is at work during AFSD is discussed next for each of the three feedstocks. CSPPs are hard and crisp compounds that are challenging to crush under high temperatures (500 °C), because of the significant softening of the Al matrix at high temperatures [42].

The high AF and AS of AA2219-casting is ascribed to the serious element segregation after casting. It is precisely because its composition segregation is the most serious, subsequently AFSD process, CSPP is broken by severe shearing, partially dissolved to the Al matrix under the action of elevated temperature, the AS of the CSPP contained in the original material is too large, based on finite diffusion rate and diffusion time, therefore, still residual CSPP, needle-like second phase disappeared. Existence of CSPP dissolution indicates that the second mechanism works. The existence of CSPP dissolution indicates that the second mechanism works. The 2219-T4 is in a solid solution state, the greatest solid solubility of copper in aluminum is 5.65%, and the copper content (6.2%) is higher than this value; therefore, there are CSPPs. Supersaturated solid solutions are in a double supersaturated state of solute atoms and crystal vacancy defects. The presence of vacancies in a solid solution promotes the diffusion of solute atoms, and this diffusion is faster at higher temperatures, leading to the formation of precipitates. During the AFSD process, the second phase cannot be dissolved, but the CSPPs are broken down into FSPPs, resulting in a decrease in AS. The heat input causes the FSPPs to continue growing, thereby increasing the AF, and allowing the first precipitation mechanism to dominate. After undergoing the AFSD process, the AA2219-T87 alloy experiences a decrease in AS and the disappearance of metastable precipitates (θ′ and θ″). Both the first and second mechanisms may work simultaneously. During the stretching process, the stress concentration near CSPPs is easily generated, which reduces the plasticity of the workpiece. To enhance the plasticity of the workpiece, it is important to minimize the size and number of CSPPs. After the AFSD process, the size of the CSPPs in the feedstock is significantly reduced and disappears, leading to an improvement in the plasticity of the AA2219 alloy.

### 4.3. Mechanical Properties

The main mechanisms for strengthening metallic materials are well known to be work hardening (also known as dislocation strengthening), solid solution strengthening, fine grain strengthening, and second phase strengthening. The relationship between yield strength and average grain size can be expressed as follows, based on the Hall–Petch formula [42]:

(5) $$\sigma_{y1}=\sigma_{0}+k\frac{1}{\sqrt{d}}\;$$

The increment in yield strength resulting from fine grain strengthening is represented by *σ_y_*. The friction stress is denoted as *σ_0_*, with a value of 20 MPa. The constant k holds a value of 0.04 MPa m^1/2^[43]. Using Equation (5), when the grain diameters are 2.3 μm, 3.1 μm and 2.0 μm, respectively, the calculated values of the yield strength increments are 23.8 MPa, 18.7 MPa, and 23.4 MPa, respectively. These values were found to be 18.7–23.8 MPa higher than the contribution of grain refinement strengthening for the feedstock.

Solid solution strengthening is achieved when the metal matrix is alloyed with other elements, and the atoms of the solute differ in size and/or shear modulus from those of the matrix. This difference in atomic characteristics results in alterations to the strain field. The ensuing local strain field affects the dislocations and impedes their movement, thereby increasing the yield strength of the material. The Fleischer equation is widely recognized as the governing factor for solid solution strengthening:

(6) $$∆\sigma_{ss}=MGb\varepsilon_{ss}^{\frac{3}{2}}\sqrt{c}\;$$

The equation in Table 3 provides the values of the symbols and the meaning. Some studies have suggested adjusting the power of c from ^1^/_2_ to 1 for nanostructured materials with grain size less than 30 nm [44]. However, since the grain size of AA2219 alloy is approximately 100 nm or larger, this adjustment is not required for this system. The value of Δ*σ_ss_*, as determined by Equation (6), depends on several factors including the concentration *c*, the difference in shear modulus between the solute and the matrix, and the size difference between the solute and solvent atoms (resulting in a lattice strain *ε*). Table 4 provides information on the difference in radii and the theoretical contributions [45] to the yield strength from these elements, for high-purity binary solid-solution alloys. In the T8-tempered condition, Mn and Cu are not in complete solid solution, as they can form second-phase precipitates or precipitates at grain boundaries. When all the solute atoms (Mn and Cu) are in solid solution and their individual effects are combined, the solid solution strengthening can reach up to 97 MPa. In AA2219-T87, many solute atoms have precipitated, resulting in a negligible contribution of solid solution to enhance the overall strength of the alloy. However, in the case of AA2219-T87 experiencing the AFSD process, most of the precipitated second phase will dissolve back into the matrix, thereby enhancing the effect of solid solution strengthening.

As the density of dislocations increases, the dislocations interact with each other and impede their own movement, thus increasing the yield strength of the metal. In this study, the Bailey–Hirsch relationship was utilized [46]:

(7) $$∆\sigma_{d}=M\alpha Gb\rho^{\frac{1}{2}}$$

The meaning and values of the symbols in Equation (7) are listed in Table 3. The dislocation density values of *ρ* were determined by XRD, using Equation 5 to calculation the dislocation strengthening incremental. The strength increments caused by source of the dislocation were calculated to be 20.5 MPa, 78 MPa, and 89.7 MPa in sample casting, T4, and T87, respectively. In contrast, dislocation strengthening contributed an increment of 64.5 MPa, 64.7 MPa and 66.3 MPa for sample casting-AFSD, T4-AFSD and T87-AFSD. Due to the large plastic deformation and heat input in the AFSD process, the material undergoes dynamic recrystallization [27] and the dislocation density remains high [47].

The increase in precipitation in the feedstock is attributed to the presence of a metastable phase. The correction effect can be explained as follows [48]:

(8) $$∆\sigma =\frac{0.85Gb}{2\pi \sqrt{1-\nu }}\frac{C}{D\left(1-\pi Ct/2D\right)}\ln\left(\frac{2D}{\pi r_{0}}\right)$$

Δ*σ* represents the incremental in yield strength attributed to precipitation strengthening, and the meaning and value of certain symbols in Equation (8) are provided in Table 3.

(9) $$C=\sqrt{fA}+\left(2/\pi -\pi /2A\right)fA$$

In Equation (8), D represents the diameter of the precipitates, f is the volume fraction of precipitates, r_0_ is the dislocation core radius (6 × 10^−10^ m) [49], and t stands for the thickness of precipitates. Calculate the f value to be 0.12 using the following formula [50]:

(10) $$\chi_{Cu}^{b}=f\chi_{Cu}^{p}+\left(1-f\right)\chi_{Cu}^{m}$$

In the equation, *χ^b^*, *χ^p^* and *χ^m^* represent the Cu concentration in the bulk alloy, precipitates, and matrix, respectively. The precipitates formed in the workpiece after the AFSD process have a disc-shaped shape. The Orowan–Ashby equation can be used to calculate the incremental increase in precipitation enhancement under these conditions.

(11) $$\Delta \sigma =\frac{0.13Gb}{\lambda }\ln\frac{r}{b}$$

In Equation (11), *λ* represents the spacing between neighboring particles and r denotes the particle radius. The meanings and values of these symbols can be found in Table 3. The measurements of *λ* and *r* were obtained from Figure 10. The formula suggests that Δ*σ* is positively correlated with *r* and negatively correlated with *λ*. The change in stress (Δ*σ*) after the AFSD process is presented in Table 5, the strengthening effect of precipitates in the workpiece after the AFSD process varies from 11 MPa. Comparing this estimate with feedstock (283.9 MPa) strength, the reduction in yield strength in the workpiece after the AFSD process is estimated to be 272.9 MPa.

The results of the calculation of the contribution of the four reinforcement mechanisms to the intensity are summarized in Table 6. After AFSD, the grain size of the feedstock in different heat treatment states is significantly reduced, leading to improved plasticity. This improvement is a result of grain size refinement and equilibrium phase (θ) refinement. The yield strength of 2219-casting is higher than that of the feedstock due to fine grain strengthening and solid solution strengthening. However, for the feedstock of 2219-T87, after AFSD, the yield strength decreases by 251.3 MPa. This decrease is due to the reduction in dislocation density and the re-dissolution of the precipitation of the second phase.

## 5. Conclusions

AFSD was performed on feedstock with different heat treatment states to investigate its broad range of applications. This study focused on the grain refinement mechanism and the behavior of second phase dissolution and precipitation after AFSD, as well as its impact on mechanical properties. The main conclusions drawn from the research are as listed below:

(1) The CDRX mechanism has demonstrated the effectiveness of the AFSD process in producing fine-grained microstructure in the AA2219 alloy, regardless of the shape of the feedstock grain. The XRD test results confirmed that even after AFSD treatment, the dislocation density in the deposition layer remains high compared to the work hardening state of feedstock.
(2) AFSD treatment was performed on the feedstock at different heat treatment stages, resulting in a workpiece with a uniform structure. The size of the θ phase in the casting-AFSD, T4-AFSD, and T87-AFSD decreased from 2.39 μm, 0.36 μm, and 0.41 μm to 0.76 μm, 0.31 μm, and 0.31 μm, respectively. This represents a comparative decrease of 68.2%, 13.8%, and 24.4%, respectively, when compared to the feedstock.
(3) The feedstock (AA2219-T87) exhibits a notable presence of primary strengthening phases (θ′ and θ″), while lacking the θ′ and θ″ phases that are found in T87-AFSD, which only contains nanoscale θ phases.
(4) The mechanical properties test demonstrated that the plasticity of the materials improved after the AFSD process due to grain refinement strengthening and θ phase refinement. The yield strength of AA2219-casting increased significantly from 79.8 MPa to 124.1 MPa after AFSD, mainly due to grain refinement and dislocation strengthening.

## Figures and Tables

**Table 1:** AA2219 nominal chemical composition.

Al	Cu	Mn	Mg	Zr	V	Zn	Ti	Fe	Si
Balance	5.8–6.2	0.2–0.4	≤0.02	0.1–0.25	0.05–0.25	≤0.10	0.02–0.1	≤0.3	≤0.2

**Table 2:** Mechanical properties of AA2219 alloy under different processing conditions.

	Ultimate Tensile Strength/MPa	Yield Strength /MPa	Elongation /%
casting	163.2 ± 0.7	79.8 ± 2.2	12.4 ± 0.3
T4	291.6 ± 1.7	151.8 ± 1.3	26.3 ± 0.2
T87	470.4 ± 0.4	398.5 ± 0.9	10.9 ± 0.1
casting-AFSD	212.3 ± 3.9	124.1 ± 3.6	29.2 ± 0.3
T4-AFSD	246.5 ± 6.9	140.4 ± 0.8	29.6 ± 0.3
T87-AFSD	257.7 ± 2.9	147.2 ± 1.1	28.4 ± 0.1

**Table 3:** Data on the primary solute atoms in AA2219 alloy and their contribution to yield strength.

Symbol	Meaning	Values	Unit
a	Lattice constant	=0.405 for fcc Al	nm
b	Magnitude of the Burgers vector	=p2/2a = 0.286 for fcc metals	nm
K	Hall–Petch constant	=0.04	Mpa m^1/2^
M	Mean orientation factor	=3.06 for the fccpolycrystalline matrix	Dimensionless
G	Shear modulus	=27.5 for Al 2219	GPa
α	Constant	=0.2 for fcc metals	Dimensionless
υ	Poisson ratio	=0.33 for Al 2219	Dimensionless
α_ε_	Constant	=2.6 for fcc metals	Dimensionless

**Table 4:** Physical meaning and values of the different symbols used in the calculation of the reinforcement mechanism.

Element	Difference in Atomic Radii (r_x_ − r_Al_)/r_Al_ (%)	Yield Strength Addition (Mpa wt.%^−1^)	Concentration (wt.%)	Contribution to Yield Strength (MPa)
Cu	−10.7	13.8	≤6.2	≤85.56
Mn	−11.3	30.3	≤0.4	≤12.12

**Table 5:** Precipitation strengthening contribution of T87-AFSD.

Material	λ (nm)	γ (nm)	Δσ (MPa)
T87-AFSD	506	76	11

**Table 6:** Estimation of intensity increments for different reinforcement mechanisms.

	Casting	T4	T87	Casting-AFSD	T4-AFSD	T87-AFSD
Δσ grain boundary (MPa)	22.6	24	24.9	46.4	42.7	48.3
Δσ solid solution (MPa)	36.7	49.8	negligible	16.2	33	21.6
Δσ dislocation (MPa)	20.5	78	89.7	61.5	64.7	66.3
Δσ orowan mechanism (MPa)	negligible	negligible	283.9	negligible	negligible	11
Experiments	79.8	151.8	398.5	124.1	140.4	147.2

## Data Availability

Data are contained within the article.
